# Mental health of helpline staff in Ukraine during the 2022 Russian invasion

**DOI:** 10.1192/j.eurpsy.2022.2306

**Published:** 2022-08-10

**Authors:** Irina Pinchuk, Ryunosuke Goto, Nataliia Pimenova, Oleksiy Kolodezhny, Anthony P. S. Guerrero, Norbert Skokauskas

**Affiliations:** 1 Institute of Psychiatry, Taras Shevchenko National University of Kyiv, Kyiv, Ukraine; 2 Department of Pediatrics, The University of Tokyo Hospital, Tokyo, Japan; 3 Department of Psychiatry, John A. Burns School of Medicine, University of Hawai’i at Mānoa, Honolulu, Hawaii, USA; 4 Regional Centre for Children and Youth Mental Health and Child Welfare - Central Norway, IPH, Norwegian University of Science and Technology, Trondheim, Norway; 5 Child and Adolescent Psychiatry Section, World Psychiatric Association (WPA), Geneva, Switzerland

Since its beginning in February 2022, the Russian invasion of Ukraine has posed numerous challenges to the Ukrainian health care system. With exposure to war, now entering its second month, Ukrainian residents are likely at increased risk of mental disorders. As such, providing health services to individuals exposed to war is essential, especially to protect their mental health [1].

Even prior to the recent invasion, Ukraine was a country with significant mental health care needs and limited resources, including the number of mental health professionals and community mental health services [2]. In March 2020, amidst health care reform-related budget cuts to mental health services [3] and the beginning of the COVID-19 pandemic, the Institute of Psychiatry of the Taras Shevchenko National University of Kyiv, Ukraine established the “Stop, Panic!” helpline to provide the Ukrainian population with confidential counseling and psychological support from qualified mental health specialists. These specialists had appropriate certifications and were experienced in managing crisis situations. The helpline has since operated 24 h a day, 7 days a week. It is free of charge and available to all residents of Ukraine. On February 25, 2022, the helpline was reorganized to provide psychological support to the Ukrainian population in the context of the Russian invasion. A week since its reorganization, the helpline joined the “Tell Me” initiative of Ukraine’s Ministry of Health, which aimed to create a platform for psychological assistance during the war. The “Stop, Panic!” helpline supplemented this platform with readily available assistance for residents of Ukraine. The team now consists of psychologists and psychiatrists from the Institute of Psychiatry and the Faculty of Psychology of Taras Shevchenko National University of Kyiv, as well as specialists from other institutions who agreed to volunteer. In an unprecedented crisis that has crippled clinical services throughout Ukraine, the helpline remains a vital form of psychological support for all residents of Ukraine.

The keystone for mental health services in humanitarian settings is mental health care providers residing in areas affected by conflict, as health care delivery from outside warzones is often challenging. However, information on the mental health of health staff in Ukraine during the Russian invasion of Ukraine has been extremely limited, given the difficulty in collecting relevant data amidst an ongoing war. We aimed to evaluate the mental health of helpline staff in Ukraine during the Russian invasion, which has continued to damage the lives of Ukrainian residents.

We conducted a cross-sectional study on helpline staff in Ukraine during the Russian invasion. Between March 18 and 26, 2022 (days 23–31 of the war), we collected data using an online self-reported questionnaire. All 27 Ukraine-based staff in the “Stop, Panic!” helpline were recruited. Information was anonymously collected on demographic characteristics, exposure to war (defined as seeing or hearing gunshots or bombings during the war), displacement of self or family, and mental health. We used multiple scales to assess the mental health of participants: the Patient Health Questionnaire-4 (PHQ-4) to screen for depression and anxiety [4], the Beck Depression Inventory (BDI) to assess the severity of depression symptoms [5], the Perceived Stress Scale (PSS) to assess stress [6], and the Maslach Burnout Inventory—Human Services Survey for Medical Personnel (MBI-HSS (MP)) to assess burnout [7]. Participants screened positive for anxiety if they scored 3 or higher in the first two questions of the PHQ-4, and they screened positive for depression if they scored 3 or higher in the last two questions of the PHQ-4 [4]. We used cutoffs for the severity of depressive symptoms as previously reported [5]. Levels of stress were expressed as a continuous variable using the total PSS score. An individual was determined to have burnout if they had either a total score of 27 or higher in the Emotional Exhaustion domain or a total score of 10 or higher in the Depersonalization domain [7]. Ethical approval was obtained from the ethics committee of Taras Shevchenko National University of Kyiv’s Institute of Psychiatry (No. 4–19032022).

We obtained data from 25 out of 27 helpline staff (response rate, 92.6%; 18 females and 7 males) based in Ukraine. A total of 15 (60%) were psychologists and 7 (28%) were physicians. Twenty-two (88%) reported being exposed to the war. Of these, 12 (48% of the total sample) reported seeing and 19 (76% of the total sample) reported hearing gunshots or bombings. None reported experiencing the death of a friend or a family member at the time of the study. Thirteen (52%) reported that their family was displaced within Ukraine and 6 (24%) reported that their family moved abroad. As for helpline staff themselves, 7 (28%) moved abroad and 13 (52%) moved within Ukraine. Based on the PHQ-4, 10 (40%) screened positive for depression and 11 (44%) screened positive for anxiety. Using the BDI, we found that 7 (28%) had mild to moderate and 4 (16%) had moderate to severe depression symptoms. The average total PSS score was 19.7 (standard deviation, 7.9). For reference, the average total PSS score was 12.1 for men and 13.7 for women in a 1983 U.S. poll, and 15.5 for men and 16.1 for women in a 2009 U.S. poll [8]. Finally, 17 (68%) had burnout, based on the MBI-HSS (MP) (Table 1).

**Table 1.** Characteristics of the helpline staff who participated in the study.

	All participants (*n* = 25)
Age (years)	39.6 ± 10.9
Gender	
Female	18 (72%)
Male	7 (28%)
Profession	
Psychologist	15 (60%)
Physician	7 (28%)
Other	3 (12%)
Exposed to war	22 (88%)
Saw gunshots, bombings, and so forth	12 (48%)
Heard gunshots, bombings, and so forth	19 (76%)
Displacement of self	
Abroad	7 (28%)
Within Ukraine	13 (52%)
Displacement of family	
Abroad	6 (24%)
Within Ukraine	13 (52%)
Stress level (PSS)	19.7 ± 7.9
Screened positive for depression (PHQ-4)	10 (40%)
Screened positive for anxiety (PHQ-4)	11 (44%)
Level of depression symptoms (BDI)	
None or minimal depression	14 (56%)
Mild to moderate depression	7 (28%)
Moderate to severe depression	4 (16%)
Severe depression	0 (0%)
Burnout (MBI-HSS (MP))	17 (68%)

*Note:* Categorical variables are expressed as number of participants (proportions) and continuous variables are expressed as mean ± standard deviation. There were two participants with missing answers to the PSS, and one participant with missing answers to the MBI-HSS (MP). Exposure to war was defined as seeing or hearing gunshots or bombings during the war.

Abbreviations: BDI, Beck Depression Inventory; MBI-HSS (MP), Maslach Burnout Inventory—Human Services Survey for Medical Personnel; PHQ-4, Patient Health Questionnaire-4; PSS, Perceived Stress Scale.

The helpline staff in our sample, nearly all of whom were exposed to the war in Ukraine, demonstrated signs of compromised mental health. They reported experiencing symptoms of depression, anxiety, and burnout, and may be at increased risk of psychopathology. Many had left their original locations of residence, a finding that may reflect the collapse of clinical services due to the shortage of staff, destruction of healthcare facilities, and limited availability of resources. As helplines may be the only source of mental health support in affected areas, these findings of compromised mental health and burnout among providers of a prominent helpline in Ukraine are indeed a cause for urgent concern.

Though the sampling design and sample size of our study may limit the generalizability of our study to all health professionals exposed to the war in Ukraine, our study indicates the need for a stronger humanitarian health care support structure in Ukraine, especially for mental health care. Helplines based outside Ukraine may help relieve the stress of those based in Ukraine, provided that the helpline staff can communicate with the Ukrainian people. International mental health organizations should work together with Ukrainian mental health organizations to make sure that the mental health needs of Ukrainian residents are met. The mental health of both health care providers and ordinary citizens in Ukraine are at stake. War must immediately come to an end.
